# Interaction and cross-talk between non-coding RNAs

**DOI:** 10.1007/s00018-017-2626-6

**Published:** 2017-08-24

**Authors:** Soichiro Yamamura, Mitsuho Imai-Sumida, Yuichiro Tanaka, Rajvir Dahiya

**Affiliations:** 1 Department of Urology, University of California, San Francisco, San Francisco, CA USA; 2 San Francisco Veterans Affairs Medical Center, San Francisco, CA USA

**Keywords:** Long non-coding RNA, Circular RNA, MicroRNA, Competing endogenous RNA, Interaction, Small nucleolar RNA, PIWI-interacting RNAs

## Abstract

Non-coding RNA (ncRNA) has been shown to regulate diverse cellular processes and functions through controlling gene expression. Long non-coding RNAs (lncRNAs) act as a competing endogenous RNAs (ceRNAs) where microRNAs (miRNAs) and lncRNAs regulate each other through their biding sites. Interactions of miRNAs and lncRNAs have been reported to trigger decay of the targeted lncRNAs and have important roles in target gene regulation. These interactions form complicated and intertwined networks. Certain lncRNAs encode miRNAs and small nucleolar RNAs (snoRNAs), and may regulate expression of these small RNAs as precursors. SnoRNAs have also been reported to be precursors for PIWI-interacting RNAs (piRNAs) and thus may regulate the piRNAs as a precursor. These miRNAs and piRNAs target messenger RNAs (mRNAs) and regulate gene expression. In this review, we will present and discuss these interactions, cross-talk, and co-regulation of ncRNAs and gene regulation due to these interactions.

## Introduction

Recent high-throughput deep sequencing has enabled genomic and transcriptomic sequencing with greater sensitivity and accuracy than the previous technologies. High-throughput RNA sequencing of whole genomes and transcriptomes have revealed that ncRNAs constitute a majority (98%) of the transcriptome with protein-coding RNAs making up the rest (2%). High-throughput sequencing has also enabled us to identify different types of non-coding RNAs (ncRNAs) and quantify their expression levels in different tissues, conditions, or developmental stages. ncRNAs have been demonstrated to have important roles in gene regulation and growing evidence indicates that non-coding RNAs interact with each other and are co-regulated.

## Long non-coding RNA–microRNA interaction

LncRNAs are transcribed RNA molecules longer than 200 nucleotides and lncRNA transcripts account for a large proportion of the non-coding transcriptome [1–3]. LncRNAs are differentially expressed in various tissues and have important functions in cellular processes such as cell proliferation, motility, and apoptosis [4–6]. LncRNAs function through diverse molecular mechanisms [7] and a number of lncRNAs associate with chromatin-modifying complexes regulating gene expression [8–12].

MicroRNAs (miRNAs) are highly conserved, single stranded, non-coding RNAs of approximately 21–24 nucleotides that regulate gene expression by post-transcriptional silencing of specific target RNAs. They repress translation or cleave RNA transcripts by binding to the 3′ untranslated region (3′UTR) of target messenger RNAs (mRNAs) through miRNA response elements (MREs) by integrating into the RNA-induced silencing complex (RISC) that contains members of the Argonaute (Ago) family of proteins which silence the integrated RNAs [13–16]. MiRNAs also regulate diverse biological processes such as cell-cycle progression, proliferation, apoptosis, and development [17, 18].

### Competitive endogenous RNA (ceRNA)

It has been reported that miRNAs bind to transcribed pseudogenes and lncRNA through MREs, which compete for the binding of these miRNAs to their mRNA-binding sites. These RNAs act as molecular sponges or decoys and suppress the targeting of mRNAs by miRNAs and thus are called competitive endogenous RNAs (ceRNAs). To be able to act as a ceRNA, an MRE in lncRNA requires incomplete complimentary to miRNA binding. Thus, the interactions of lncRNAs with miRNAs do not trigger decay of the interacting RNAs or trigger only slow decays. CeRNA activity regulates diverse cellular processes in development and diseases (Table 1), and lncRNA-miRNA interactions form intertwined and complex regulatory networks. Pioneering works has been published in plants for (lncRNA), human cells (pseudogenes), and viruses (small ncRNAs) as described below.

**Table 1:** Non-coding RNA partners in ceRNA regulatory mechanism

Non-coding RNA	miRNA	mRNA targets of miRNA	Function	Human disease, species, etc.	References
Pseudogene					
PTENP1	miR-20a, miR-19b, miR-21, miR-26a and miR-214	PTEN	Tumor suppression	Human	[20]
KRAS1P	miR-143 and let-7 family	KRAS	Carcinogenesis	Human	[20]
PTENP1	miR-17, miR-19b and miR-20a,	PTEN	Tumor suppression	Hepatocellular carcinoma	[21]
Pbcas4	miR-185	Bcl2, Il17rd, Pnpla3, Shisa7 and Tapbp		Mouse and human	[22]
HMGA1P7	miR-15, miR-16, miR-214 and miR-761	H19 and Igf2	Carcinogenesis	Mouse	[23]
Small ncRNA					
HSURs 1 and 2	miR-142-3p, miR-27 and miR-16	FOXO1	Viral infection	Human T cells	[24]
Circular RNA					
ciRS-7 (CDR1as)	miR-7		Brain development	Human and mouse	[40, 41]
		Myrip	Diabetes suppression	Human and mouse	[42]
Sry	miR-138			Mouse	[40]
lncRpa and circRar1	miR-671	CASP8 and P38	Apoptosis	Mouse neuronal cell	[110]
circHIPK3	miR-124	IL6R and DLX2	Carcinogenesis	Human	[43]
circRNA-CER	miR-136	MMP13	Osteoarthritis	Human chondrocytes	[111]
lncRNA					
IPS1	miR-399	PHO2	Plant growth	*Arabidopsis thaliana*	[19]
linc-ROR	miR-145	Oct4, Nanog and Sox2	Self-renewing	Human embryonic stem cell	[28]
	miR-205	ZEB2	Carcinogenesis	Breast cancer	[26]
HOTAIR	miR-331-3p	HER2	Carcinogenesis	Gastric cancer	[32]
	miR-141	SKA2	Carcinogenesis	Glioma	[112]
MALAT1	miR-124	GRB2	Carcinogenesis	Cervical cancer	[113]
H19	miR-106a	CDKN1A, DICER1, RB1, ARID4B, ANKRD52 and FAM102A	Myoblast differentiation	Human myoblast	[38]
	miR-138 and miR-200a	Vimentin, ZEB1, and ZEB2	Carcinogenesis	Hepatocellular carcinoma	[108]
	miR-200b/c and let-7b	Git2 and Cyth3	Carcinogenesis	Breast cancer	[114]
HULC	miR-372	PRKACB	Carcinogenesis	Liver cancer	[44]
linc-MD1	miR-133 and miR-135	MAML1 and MEF2C	Myoblasts differentiation	Mouse and human myoblasts	[45]
BACE1-AS	miR-485-5p	BACE1	Alzheimer’s disease	Human brain	[46]
lncRNA-ATB	miR-200 family	ZEB1 and ZEB2	Carcinogenesis	Hepatocellular carcinoma	[47]
CCAT1	miR-218-5p	Bmi1	Carcinogenesis	Gallbladder cancer	[115]
ZFAS1	miR-150	ZEB1, MMP14 and MMP16	Carcinogenesis	Hepatocellular carcinoma	[116]
lncRNA-BGL3	miR-17, miR-93, miR-20a, miR-20b, miR-106a and miR-106b	PTEN	Tumor suppression	Leukemia	[117]

#### CeRNA in plants

The first ceRNA reported was lncRNA IPS1 in plants. In *Arabidopsis thaliana*, the lncRNA IPS1 has a binding site for the phosphate starvation-induced miR-399 [19]. The mismatched sequence at the potential miRNA-binding site in IPS1 prevents miR-399 from cleaving IPS1. Therefore, IPS1 acts as a ceRNA for PHO2 that encodes an E2 ubiquitin conjugase-related protein, a target of miR-399 [19].

#### Pseudogene ceRNA

Pseudogenes are genomic DNA sequences similar to normal genes but do not express their gene. The non-coding PTENP1 pseudogene is highly homologous to the tumor suppressor, phosphatase, and tensin homolog (PTEN). Poliseno et al. have reported that the 3′UTR of PTENP1 is targeted by multiple miRNAs (miR-20a, miR-19b, miR-21, miR-26a, and miR-214) that also target the 3′UTR of PTEN. Therefore, PTENP1 functions as a ceRNA (sponge) for PTEN by preventing its repression by these miRNAs. Thus, the 3′UTR of PTENP1 functions as a tumor suppressor [20]. In the normal tissue and prostate tumor samples, the correlation between PTEN and PTENP1 expression was found, suggesting ceRNA regulation in these genes [20]. This study also describes that the 3′UTR of KRAS1P pseudogene has binding sites for miR-143 and the let-7 miRNA family, and acts as a ceRNA for KRAS, thereby functioning as an oncogene [20]. This was the first study to describe that pseudogenes have function and act as ceRNAs. However, the binding sequences in PTENP1 3′UTRs perfectly match the sequences of the miRNA seed regions which are contrasting to ceRNA regulation by IPS1 (1.1.1) and circular RNA *ciRS*-*7* (*CDR1as*) (1.1.5). These binding may also trigger a decay of PTENP1.

Also PTENP1 decoys oncogenic miRNAs, miR-17, miR-19b, and miR-20a, and reduces downregulation of their target gene, PTEN in hepatocellular carcinoma [21]. PTENP1 also rescues PH domain and leucine-rich repeat protein phosphatase (PHLPP, a negative AKT regulator), autophagy proteins Unc-51 like autophagy activating kinase 1 (ULK1), autophagy-related 7 (ATG7), and sequestosome 1 (p62), from downregulation by the miRNAs [21]. The mouse Pbcas4 pseudogene functions as a ceRNA for human breast carcinoma amplified sequence 4 (BCAS4) by sponging miR-185 and suppressing the repression of miR-185 target genes, Bcl2, Il17rd, Pnpla3, Shisa7, and Tapbp [22]. LncRNA H19 and insulin like growth factor 2 (Igf2) are targeted by high mobility group AT-hook 1 pseudogene 7 (HMGA1P7)-targeting miRNAs (miR-15, miR-16, miR-214, and miR-761), and thus, HMGA1P7 functions as a ceRNA for H19 and Igf2 in mice [23]. In this study, widely used overexpression and knockdown were also applied, which may lead to un-physiological conditions. However, HMGA1P7, H19, and IGF2 expression was found to clearly positively correlate in human breast cancer samples [23], indicating that the ceRNA regulation may potentially occur under physiological condition.

#### Small ncRNA ceRNA

T cells transformed by Herpesvirus saimiri express small non-coding RNAs called viral U-rich non-coding RNAs (HSURs). HSURs 1 and 2 were found to have potential binding sites for three host-cell miRNAs, miR-142-3p, miR-27, and miR-16. Coimmunoprecipitation shows that HSURs 1 and 2 interact with these miRNAs in T cells transformed by Herpesvirus saimiri. HSUR1 induces degradation of miR-27 in a sequence-specific manner and prevents reduction of expression of forkhead box 1 (FOXO1), a target of miR-27 [24]. This indicates that viral small ncRNAs have potential to manipulate host cells through ceRNA mechanisms. These results indicate that triggering decay of miRNA elicits ceRNA regulation, which is contrasting to other ceRNA mechanisms by which lncRNA are claim to only sequester miRNAs.

#### lncRNA ceRNA

##### LincRNA-RoR ceRNA

LincRNA-RoR was identified as a lincRNA whose expression was increased in induced pluripotent stem cells (iPSCs) compared with embryonic stem cells (ESCs) using microarray [25]. It may inhibit cellular stress pathways through the p53 response and promote survival in iPSCs and ESCs [25]. This is the first report which demonstrates that lincRNAs are capable of reprogramming ESCs to iPSCs [25]. LincRNA-RoR has also been reported to function as an oncogene [26, 27]. In stem cell field, an important ceRNA function of linc-RoR was reported. Linc-RoR has binding sites for miR-145 which binds to the 3′UTR of transcription factors, Oct4, Nanog, and Sox2 [28]. Linc-RoR functions as a ceRNA to prevent degradation of these transcription factors by miR-145 targeting, and regulates transcriptional and epigenetic networks in human embryonic stem cell [28].

##### HOTAIR ceRNA

HOTAIR is an lncRNA which is localized in the Homeobox C (HOXC) gene cluster on chromosome 12. It interacts with the polycomb repressive complex 2 (PRC2) and lysine-specific demethylase 1 (LSD1) complex, enhancing H3K27 trimethylation and H3K4 demethylation, respectively, to suppress expression of multiple genes [29]. HOTAIR has been well studied and has been shown to promote cancer cell invasiveness [29, 30] and to increase cell proliferation, cell-cycle progression, and reduce apoptosis [31]. HOTAIR acts as a ceRNA for human epithelial growth factor receptor 2 (HER2) by binding to miR-331-3p to prevent degradation of HER2, a target of miR-331-3p in gastric cancer [32]. In this study, HOTAIR was also shown to be a target of miR-124 [32]. This is the first HOTAIR ceRNA study and made another milestone in HOTAIR research.

##### LncRNA H19 ceRNA

LncRNA H19 is one of the most extensively studied lncRNAs. H19 is highly expressed maternally in the developing mouse embryo with the adjacent insulin like growth factor 2 (Igf2) gene being transcribed from the paternal allele [33]. H19 is only abundant in skeletal muscle after birth [33], and H19 is developmentally regulated and is activated very early during muscle cell differentiation [34]. H19 has been reported to be upregulated and promoted various cancers [35]; however, tumor suppressing effects of H19 has also been reported [36, 37].

H19 ceRNA activity in myoblast differentiation has been reported. MiR-17-5p family members including miR-106a were found to bind H19 in HeLa cells and myoblasts using miRNA crosslinking and immunoprecipitation (miR-CLIP) as described in 1.4. During myoblast differentiation, H19 level increases and level of miR-17-5p family members decreases, suggesting that H19 acts as a ceRNA for these miRNAs. Overexpression of miR-106a and H19 RNAi also supported the ceRNA activities [38].

#### Circular RNA ceRNA

Circular RNA (circRNA) is a non-coding loop RNA in which the 3′ and 5′ ends have been joined together. CircRNAs are resistant to endonuclease enzymatic degradation, since they do not have susceptible 5′ and 3′ ends; therefore, circRNAs are more stable than linear RNAs. The sex determining region Y (SRY) ncRNA was the first-reported mammalian circRNA which was found in mouse testis. Investigation of circRNAs is an early stage, although SRY was discovered about 25 years ago [39].

**Fig. 1:**
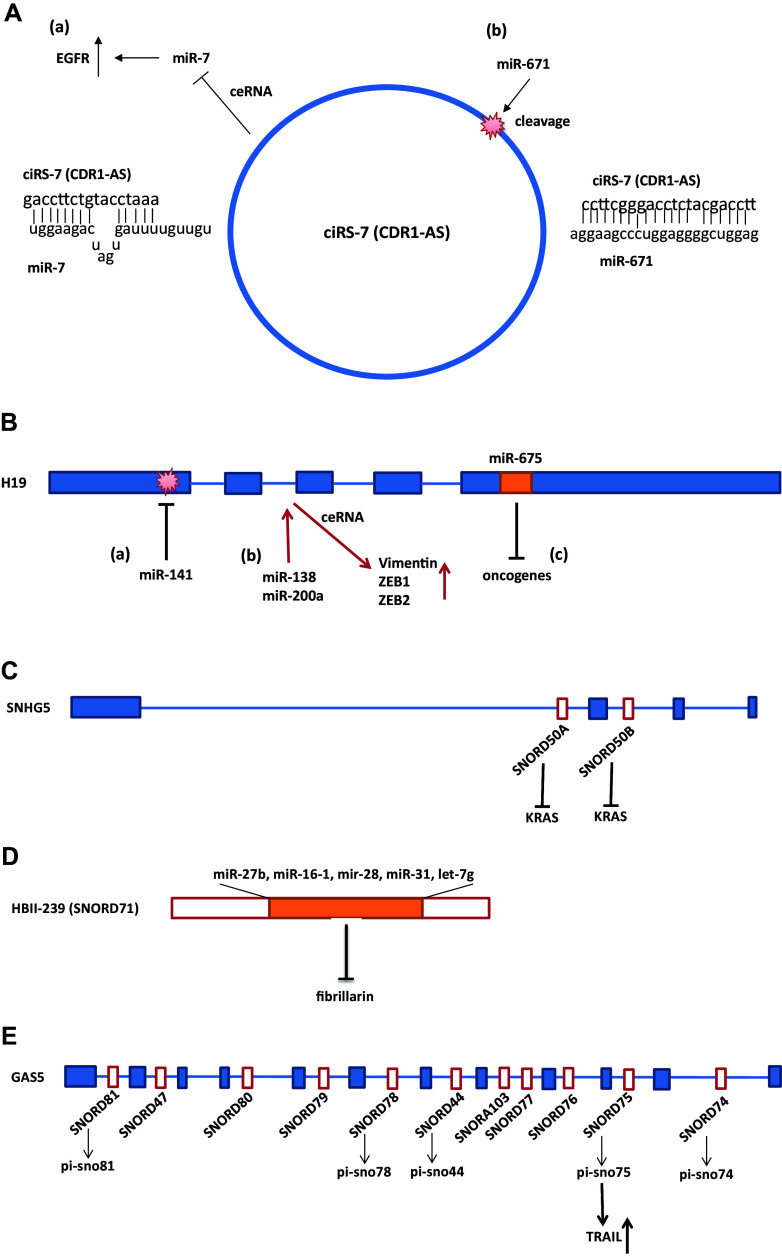
Representative interactions and cross-talk between non-coding RNAs. **A** Interaction and co-regulation between circular RNA, ciRS-7 (CDR1as), and miRNAs. (*a*) ciRS-7 (CDR1as) contains more than 60 binding sites for miR-7. A mismatch at the central part of the binding region prevents miRNA-mediated cleavage. Thus, ciRs-7 acts as a sponge for miR-7 and increase levels of miR-7 targets [40, 41]. (*b*) MiR-671 binds to ciRS-7 in a sequence-specific manner and suppresses ciRS-7 expression and function [48]. **B** Interactions and co-regulation between lncRNA H19 and miRNAs. (*a*) MiR-141 binds to H19 in a sequence-specific manner and suppresses H19 expression and its oncogenic function [107]. (*b*) H19 functions as a ceRNA for miR-138 and miR-200a, and reduces suppression of their targets Vimentin, ZEB1, and ZEB2 [108]. (*c*) MiR-675 which is encoded by H19 targets mRNAs of oncogenes and functions as a tumor suppressor [82, 109]. **C** LncRNA SNHG5 encodes SNORD50A and SNORD50B which inhibit KRAS function. Expression of these snoRNAs may be dependent on the host lncRNA, SNHG5, and expression [90]. **D** HBII-239 (SNORD71) encodes miRNAs. Expression of these miRNAs may depend on the expression of the host snoRNA, HBII-239 (SNORD71). The C/D box snoRNA HBII-239 (SNORD71)-derived miRNA precursors bind to fibrillarin protein, a component of a nucleolar small nuclear ribonucleoprotein (snRNP) [94]. **E** LncRNA GAS5 encodes snoRNAs that generate piwi-interacting RNAs, piRNAs. Pi-snoRNA 75, a piRNA, activates tumor necrosis factor (TNF)-related apoptosis inducing ligand (TRAIL) [97]

Notable ceRNA studies of circRNA were reported for cerebellar degeneration-related protein 1 (CDR1) antisense transcript, ciRS-7 (CDR1as). CiRS-7 contains more than 60 selectively conserved target sites for miR-7 [40, 41]. A mismatch at the central part of the target sites prevents miRNA-mediated cleavage; thus, ciRs-7 acts as a sponge for miR-7 and increases levels of miR-7 targets (Fig. 1A) [40, 41]. The high number of miR-7 target sites in ciRS-7 may have an advantage for eliciting ceRNA regulation. SRY was also found to function as a miR-138 sponge [40]. The ceRNA functions of CiRS-7 in diabetes have also been examined. MiR-7 targets myosin VIIA and Rab interacting protein (Myrip) which stimulates insulin release from plasma membrane and paired box 6 (Pax6), a transcription factor that activates insulin transcription in pancreatic β cells. CiRS-7 sponges miR-7 to improve β cell function by rescuing Myrip and Pax6 from their downregulation in diabetes [42].

Recent study shows that circHIPK3, circRNA derived from the HIPK3 gene Exon2, was found to be significantly abundant in various tissues compared with HIPK3 and upregulated in liver cancer compared with matched normal tissues [43]. The knockdown of circHIPK3 significantly inhibits human cell growth. CircHIPK3 was found to bind to 9 miRNAs with 18 potential binding sites [43]. Because of these high number of binding sites, circHIPK3 may potentially have ceRNA function under natural conditions. In particular, it has been demonstrated that circHIPK3 functions as a ceRNA for miR-124 and inhibits miR-124 targeting of proliferation-promoting genes, IL6R and DLX2 [43].

##### Other lncRNA ceRNA

Computational analysis has shown that lncRNA HULC contains miR-372-binding sites [44]. HULC may act as a ceRNA, downregulating the activity of miR-372 which downregulates protein kinase C alpha (PRKACB) translation and induces phosphorylation of CREB in liver cancer [44].

Linc-MD1 was found to be expressed during myoblast differentiation, host precursors of muscle-specific miRNAs, miR-206, and miR-133b. Linc-MD1 contains two miR-135 binding sites and one for miR-133. MiR-133 and miR-135 downregulate the expression of their target genes, mastermind like transcriptional coactivator 1 (MAML1), and myocyte enhancer factor 2C (MEF2C), that activate muscle-specific genes in mouse and human myoblasts. Linc-MD1 acts as a ceRNA by binding to miR-133 and miR-135, and promotes muscle differentiation [45]. A pre-miR-133b hairpin structure in linc-MD1 may limit miR-133 accessibility and the smaller number of the binding sites could limit ceRNA activity, which is contrasting to ciRS-7 ceRNA activity.

LncRNA has been show to involve in Alzheimer’s disease. LncRNA BACE1-AS, which is highly upregulated in brain samples from Alzheimer’s patients, acts as a ceRNA for beta-Secretase 1 (BACE1) mRNA by binding to miR-485-5p. A mismatch at the center of the binding site prevents downregulation of BACE1 mRNA, a target of miR-485-5p, which was demonstrated by knockdown of miR-485-5p and BACE1-antisense overexpression experiments [46].

Transforming growth factor beta (TGF-b)-inducing lncRNA-ATB functions as a decoy for the miR-200 family, which upregulates ZEB1 and ZEB2, targets the miR-200 family and induces EMT and invasion in hepatocellular carcinoma [47]. However, lncRNA-ATB binds to the miR-200 family through the perfect-matched seed regions of the miRNAs, which may also decay lncRNA-ATB.

### MiRNA targeting of lncRNA

An increasing number of publications demonstrate that miRNAs interact with lncRNAs through MRE, thereby triggering decay of lncRNA or repressing its function (Table 2). The first evidence for an ncRNA as a miRNA target was the finding that MiR‐671 targets and cleaves circRNA, the cerebellar degeneration-related protein 1 (CDR1) antisense transcript, and ciRS-7 (CDR1as) in an Ago2‐dependent manner in human cells [48]. CiR-7 (CDR1as) was found to be formed via non-linear splicing [48]. There is positive correlation between the cerebellar degeneration-related protein 1 (CDR1) mRNA and ciR-7 (CDR1as) expression levels [48]. The decrease of ciR-7 (CDR1as) levels by miR-671 also reduces the CDR1 mRNA levels [48]. As described in 1.1.5, ciRS-7 functions as a ceRNA for miR-7 and also as a target of miR-671 leading to cleavage, which depends on the binding sequences between ciRS-7 and these miRNAs. Cleavage requires perfect or near perfect match of the binding site, while ceRNA mechanisms require mismatches in the binding sequences not to cleave the target (Fig. 1A).

**Table 2:** MiRNA targeting of lncRNA by direct interaction

LncRNA	miRNA	mRNA targets of miRNA	Human disease, species, etc.	References
circRNA ciRS-7 (CDR1as)	miR‐671		Human cells	[48]
Oncogenic lncRNA				
HOTAIR	miR-130a-3p		Gallbladder cancer	[67]
	miR-141		Kidney cancer	[118]
	miR-152	HLA-G	Gastric cancer	[119]
	miR-34a		Prostate cancer	[120]
	miR-326	FGF1	Glioma	[121]
	miR-1		Hepatocellular carcinoma	[122]
UCA1	miR-143		Breast cancer	[123]
	miR-507	FOXM1	Melanoma	[124]
MALAT1	miR-217		Lung cancer	[125]
	miR-125b	SIRT7	Bladder cancer	[126]
	miR-9		Hodgkin lymphoma and glioblastoma	[49]
	miR-9		Osteosarcoma	[127]
lincRNA-p21	let-7		Cervical carcinoma	[128]
H19	miR-141		Gastric cancer	[107]
TreRNA	miR-190a		Hepatoma	[129]
ENST00000515084	miR-370		Breast cancer	[130]
HOTTIP	miR-125b		Hepatocellular carcinoma	[131]
	miR-192 and miR-204		Hepatocellular carcinoma	[50]
lncRNA AC130710	miR-129-5p		Gastric cancer	[132]
lincRNA NR_024015	miR-526b		Gastric cancer	[133]
PCGEM1	miR-145		Prostate cancer	[134]
XIST	miR-152		Glioblastoma	[135]
UFC1	miR-34a	CTNNB1	Hepatocellular carcinoma	[136]
Tumor suppressor lncRNA				
loc285194	miR-211		Colon cancer	[137]
GAS5	miR-21		Breast cancer	[138]
TUSC7	miR-23b		Gastric cancer	[139]
CASC2	miR-21		Glioma	[140]
uc003opf.1	miR-149-3p		Esophageal squamous cell carcinoma	[141]
Muscle differentiation				
H19	let-7 miRNA family		Human and mouse	[142]
Neurodegeneration				
lnc-SCA7	miR-124	SCA7	Mouse	[51]
Computer analysis				
PVT1 (ceRNA)	miR-200 family		Normal breast tissues	[143]
FERIL4 (ceRNA)	miR-106a-5p	PTEN, RB1, RUNX1, VEGFA, CDKN1A,		
		E2F1, HIPK3, IL-10, PAK7 and RB1	Gastric cancer	[144]
7sl RNA	miR-125b		Zebra fish	[145]
STXBP5-AS1 (ceRNA)	miR-190b	ERG, STK38L and FNDC3A	Breast cancer	[146]

LncRNA metastasis associated with lung adenocarcinoma transcript 1 (MALAT1) was originally found to be highly expressed in metastatic non-small-cell lung cancers and is a highly conserved lncRNA [102]. MALAT1 is upregulated in various cancers, and promotes proliferation and metastasis of tumor cells. MiR-9 targets lncRNA MALAT1 through its binding site in an Ago2-dependent manner in the nucleus which was revealed by in situ hybridization and confocal microscopy [49]. This study clearly shows that the miRNA interacts with the lncRNA in the nucleus. MiR-192 and miR-204 were found to target HOTTIP in a sequence-specific and Ago2-dependent manner and inhibited proliferation of hepatocellular carcinoma [50]. Microarrays identified glutaminase (GLS1), whose marked elevation in cancers has been observed as a downstream gene of HOTTIP [50]. In tissue specimens, miR-192 and miR-204 expressions are low and HOTTIP is high in hepatocellular carcinoma compared with normal tissues, showing a negative correlation between these miRNAs and HOTTIP expression [50]. This study indicates that interaction between lncRNA and miRNA is highly relevant to cancer progression.

In neurodegenerative disorders, interaction between miRNA and lncRNA has been reported. A CAG repeat expansion in ATXN7, an essential component of the mammalian transcription coactivator complex, STAGA, causes spinocerebellar ataxia type 7 (SCA7), a neurodegenerative disorder. STAGA induces miR-124 which binds to the 3′UTR of lnc-SCA7 and ATX7, a component of STAGA, and suppresses the expression of these genes in a sequence-specific manner, showing intriguing feedback regulation [51].

### Computational analysis of lncRNA–miRNA interaction

There have been a number of reports that demonstrate lncRNA–miRNA interaction identified by computational or data analysis (Table 2). The algorithms for miRNA target search are based on conserved, six-nucleotide interactions between the 5′ end of the miRNA, the seed region, and the 3′UTR of the target mRNA. Since the miRNA target is predicted experimentally and computationally using various algorithms, the results vary in these different algorithms [52]. Bioinformatic prediction of lncRNA-miRNA and ceRNA interactions depends on the algorithms used for miRNA target search; therefore, varied predictions are produced with different algorithms.

### Experimental miRNA–lncRNA interactions: methodology and data base

Experimental validation is indispensable for documenting miRNA–lncRNA interactions and ceRNA mechanisms. Crosslinking immunoprecipitation (CLIP) with antibodies against components of the RNA-induced silencing complex (RISC), particularly the Argonaute (AGO) family, has been developed and widely used for RNA interaction studies. CLIP sequencing (CLIP-Seq) techniques, an application of high-throughput sequencing methodologies, have been used extensively to characterize biologically relevant protein–RNA interactions [40].

One of the important methods in miRNA–lncRNA interaction study, miRNA crosslinking, and immunoprecipitation (miR-CLIP), was developed with H19. In this method, miRNA with psoralen and biotin are transfected into cells. After photo-crosslinking, Ago2 immunoprecipitation is performed, followed by streptavidin affinity purification of the miRNA-linked RNAs. The captured miRNA targets are then identified by deep sequencing. MiR-CLIP with pre-miR-106a, a miR-17-5p family member, was performed and H19 was identified as an miR-106 target. MiR-106a targets which TargetScan indicated were significantly enriched by miR-CLIP compared to non-targets, verifying that the method was effective [38].

CLIP-Seq-based approaches have been applied to different types of cells and tissues in RNA research. Advanced CLIP-Seq techniques such as (1) high-throughput sequencing of RNA isolated by crosslinking immunoprecipitation (HITS-CLIP) [53, 54]; (2) photoactivatable-ribonucleoside-enhanced crosslinking and immunoprecipitation (PAR-CLIP) [55]; and (3) crosslinking, ligation, and sequencing of hybrids (CLASH) [56] are able to identify a large number of Argonaute-bound target sequences that contain miRNA-binding sites in targeted RNAs. The databases have been developed by integrating these data and the public data resources for interactions between lncRNA and miRNA and ceRNA as listed in Tables 3 and 4, respectively.

**Table 3:** LncRNA–miRNA databases

Database	URL	Contents	References
DIANA-LncBase v2	http://www.microrna.gr/LncBase	Database provides two different miRNA–lncRNA interaction modules. One module is experimentally supported, and the other is in silico predicted interactions Database is supported by low-yield experimental techniques, analysis of more than 150 CLIP-Seq libraries, and publications	[57, 58]
LNCediting	http://bioinfo.life.hust.edu.cn/LNCediting/	Database provides information about RNA editing in lncRNAs. To predict the functional changes after RNA editing, it shows changes in the secondary structure and miRNA–lncRNA interactions	[59]
NPInter v3.0	http://www.bioinfo.org/NPInter/	Database provides information about interactions between ncRNAs (except tRNAs and rRNAs), lncRNAs and others. It offers various types of basic information about the interaction Database is supported by in silico predictions from AGO CLIP-Seq, 991 publications, and high-throughput technologies	[60–62]
lncReg	http://bioinformatics.ustc.edu.cn/lncreg/	Database is derived from miRNA–lncRNA interaction information in 259 research articles Database provides basic information about lncRNA, genes, relationships, mechanisms from 1,081 entries, and lncRNA-related regulatory networks	[63]
LNCipedia v4.0	http://www.lncipedia.org	Database has comprehensive human lncRNA information which contains RNA sequence, structure, local conservation, and transcript information Database also offers information about possible miRNA–lncRNA interactions. This prediction analysis is supported using the Mir Target2 algorithm	[63, 65]
lncRNASNP	http://bioinfo.life.hust.edu.cn/lncRNASNP/	Prediction of effects on SNPs in lncRNA secondary structure and lncRNA–miRNA interaction	[66]
starBase v2.0	http://starbase.sysu.edu.cn/	Database provides comprehensive interaction networks of ncRNAs (lncRNAs, miRNAs, and ceRNAs), mRNA, and RNA-binding proteins in normal tissues and cancer cells based on 108 CLIP-Seq	[68]
LncRNAMAP	http://lncRNAMap.mbc.nctu.edu.tw/	Database of putative regulatory functions of lncRNAs Database is supported by publicly available deep sequencing data	[69]

**Table 4:** CeRNA databases

Database	URL	Content	References
spongeScan	http://spongescan.rc.ufl.edu	Database predicts miRNA response elements in lncRNAs and presumes lncRNA function as miRNA sponges Sequence complementarity underlies this database	[70]
SomamiR 2.0	http://compbio.uthsc.edu/SomamiR	Database contains functional analysis of expected miRNA–ceRNA (including mRNAs, circular RNAs and lncRNA) interaction/changes caused by somatic mutations in cancer	[71, 72]
ceRNABase	http://starbase.sysu.edu.cn/mrnaCeRNA.php	Part of starBase v2.0	[68]
ln*Ce*DB	http://gyanxet-beta.com/lncedb/	Database of human lncRNAs that act as ceRNAs. This database assesses lncRNA–mRNA interactions which are potentially controlled by common miRNAs	[73]

### Overlapping of ceRNA regulatory mechanisms and downregulation of lncRNA by direct miRNA–lncRNA interactions

The interactions between lncRNAs and miRNAs which mediate ceRNA mechanisms may trigger silencing of lncRNAs under certain conditions, since lncRNAs may be integrated into the RISC complex and potentially cleaved by Argonaute proteins. The cleavage requires perfect or near perfect match of the binding site. CeRNA mechanisms require mismatches in the binding sequences to avoid immediate cleavage of the target. If the binding is long enough, it may trigger decay, although no information about requirement for binding duration is available. The majority of ceRNA studies show perfect or near perfect matches of biding sequences between lncRNAs and miRNAs. Therefore, these reported ceRNA interactions may also lead to cleavage of lncRNAs in addition to ceRNA regulation. In fact, viral small ncRNA HSUR1 induces degradation of miR-27 in a sequence-specific manner and functions as a ceRNA [24]. Every interaction between miRNA and lncRNA may trigger decay of lncRNA or inhibit lncRNA function and also may function as ceRNA. Therefore, these two mechanisms may overlap each other.

### Indirect interaction and co-regulation between lncRNA and miRNA

A number of reports indicate that inverse expression levels between miRNA and lncRNA may occur without direct binding, suggesting cross-talk between these ncRNAs (Table 5).

**Table 5:** Indirect interaction between lncRNA and microRNA

LncRNA	miRNA	mRNA targets of miRNA	Human disease, species, etc.	References
Oncogenic lncRNA				
UCA1	miR-143		Bladder cancer	[147]
HULC	miR-9	PPARA	Hepatoma	[74]
HOTAIR	miR-125a-5p	CASP2	Colon cancer	[148]
	miR-7	SETDB1	Breast cancer stem cells	[149]
	miR-568	NFAT5	Breast cancer	[150]
H19	miR-141		Osteoblasts	[151]
BANCR	miR-9	NF-kB1	Gastric cancer	[152]
ANRIL	miR-99a and miR-449a		Gastric cancer	[153]
ncNRFR	let-7		Mouse colonic epithelia	[154]
CCAT2	miR–17–5p and miR–20a		Colon cancer	[75]
Tumor suppressor lncRNA				
MEG3	miR-29a and miR-185	DNMT1, 3A and 3B	Hepatocellular carcinoma	[155]
	miR-29a	DNMT1 and 3B	Hepatocellular carcinoma	[156]
	miR-148a	DNMT1	Gastric cancer	[157]
	miR-181b	12/15-LOX	Mouse	[158]

Interestingly, lncRNA HULC was found to regulate lipid metabolism. HULC upregulates the transcriptional factor peroxisome proliferator-activated receptor alpha (PPARA) which increases acyl-CoA synthetase long-chain family member 1 (ACSL1), leading to triglycerides and cholesterol production and proliferation of hepatoma cells [74]. Cholesterol upregulates HULC expression through the retinoid receptor RXRA, which also increases HULC expression by activating the HULC promoter, indicating a positive feedback [74]. HULC also increases methylation of CpG islands in the miR-9 promoter and suppressed miR-9 targeting of PPARA. Thus, HULC promotes lipogenesis and malignancy in hepatoma cells [74].

Single-nucleotide polymorphisms (SNP) in lncRNA were reported to affect its function. LncRNA CCAT2 which contains the colorectal cancer-risk-associated rs6983267 SNP transcriptionally upregulates MYC, miR-17-5p, and miR-20a by physically interacting with TCF7L2. This activates the WNT signaling pathway and causes metastatic progression and chromosomal instability in colon cancer [75]. CCAT2 is also a WNT downstream target, suggesting the existence of a feedback loop [75]. The rs6983267 G allele was found to produce more CCAT2 transcript than T allele [75].

## Non-coding RNA as precursors for shorter non-coding RNA

High-throughput deep sequencing of transcriptomes shows that certain lncRNAs encode miRNAs and other ncRNAs encode shorter ncRNAs which is discussed below. The expression of these ncRNAs may be correlated.

### LncRNA encoding miRNA

Microarray profiling of miRNAs has demonstrated that miRNAs are frequently coexpressed with their host genes [76]. In addition, miRNAs have been found to be located in the exons of ncRNAs and the introns of protein-coding genes [77]. For example, antiPeg11 (antiRtl1) encodes miR-431, miR-433, miR-127, miR-432, and miR-136 [78, 79]. Thus, lncRNAs may regulate their encoding miRNAs as precursors (Table 6).

**Table 6:** LncRNA encoding miRNA

LncRNA	miRNA	mRNA targets of miRNA	Human disease, species, etc.	References
antiPeg11 (antiRtl1)	miR-431, miR-433, miR-127, miR-432 and miR-136		Mouse	[78, 79]
Oncogenic lncRNA				
H19	miR-675		Mouse myoblast	[80]
		RUNX1	Gastric cancer	[159]
		RUNX1	Gastric cancer	[160]
			Human cancers	[81]
		CALN1	Gastric cancer	[161]
		c-Cbl and Cbl-b	Breast cancer	[162]
		RB	Colorectal cancer	[163]
		CDH13	Glioma cell	[164]
LncRNA Ftx	miR-374b/421 and miR-545/374a		Hepatocellular carcinoma	[84]
NCR143/145	miR-143 and miR-145		Human cancer	[85]
LOC554202	miR-31		Breast cancer	[165]
MONC	miR-125b-2, miR-99a and let-7c		Acute megakaryoblastic leukemia	[166]
MIR100HG	miR-100, miR-125b-1 and let-7a-2		Acute megakaryoblastic leukemia	[166]
Tumor suppressor lncRNA				
H19	miR-675	Igf1r	Mouse embryonic and trophoblast stem cells	[82]
		TGFBI	Prostate cancer	[109]
LINC00478	Let-7c, miR99a and miR125b	HER2	Breast cancer	[83]

### LncRNA H19 encodes miR-675

H19/miR-675 function has been well studied and established. H19 exon1 encodes miRNAs, miR-675-3p, and miR-675-5p, which are induced during skeletal muscle differentiation [80]. Reintroduction of miR-675-3p and miR-675-5p rectifies abnormal skeletal muscle regeneration after injury in H19-deficient mice [80]. miR-675-3p and miR-675-5p promote skeletal muscle differentiation and regeneration by targeting the anti-differentiation Smad transcription factors [80]. This study clearly demonstrated the H19/miR675 role in vivo.

H19/miR-675 has been reported to be an oncogene. TGF-β and hypoxia were shown to induce H19, miR-675, and EMT markers such as Snail and Slug. TGF-β induces Slug, H19, and miR-675 through the PI3K/AKT pathway, while H19 induces Slug and suppresses E-cadherin in cancer cells [81]. H19/miR-675 has also been reported to be a tumor suppressor. The release of miR-675 from H19 is inhibited by the stress-response RNA-binding protein, ELAV Like RNA-Binding Protein 1 (HuR) [82]. MiR-675 potentially targets growth-promoting Igf1r and inhibits proliferation of embryonic and extra-embryonic cell lines [82]. Both tumor suppressor and oncogenic functions have been reported for H19/miR-675, suggesting that its function depends on cell type.

### Other lncRNAs which encode miRNAs

MiRNA profiling shows that let-7c and miR125b are encoded in an intron of the lncRNA LINC00478, and are found to be elevated in the estrogen-dependent human breast cancer cell line compared with its estrogen-independent derivative [83]. In breast cancer cell lines, miR125b and let-7c directly target the HER2 3′UTR, downregulating HER2, an oncogene which has been shown to play an important role in the progression of breast cancer [83]. LncRNA Ftx hosts the miR-374b/421 and miR-545/374a clusters in its intron. The miR-545/374a cluster was found to be upregulated in Hepatitis B virus (HBV)-related hepatocellular carcinoma tissues in comparison to matched non-cancerous liver tissue specimens. Overexpression and knockdown of miR-545/374a show that the miR-545/374a cluster may promote tumorigenesis of HBV-related hepatocellular carcinoma [84]. DEAD-box RNA helicase 6, DDX6 (p54/RCK), which is accumulated in processing bodies (P-bodies), promotes the degradation of lncRNA NCR143/145 RNA, a host gene of tumor suppressors miR-143/145. The post-transcriptional downregulation of miR-143/145 promotes malignancy in cancer cells [85]. These studies including H19/miR-675 indicate that the miRNAs encoded in lncRNA have functional roles.

### LncRNA encoding small nucleolar RNA

Small nucleolar RNAs (snoRNAs) are non-coding RNAs of approximately 60–200 nucleotides, which chemically modify other RNAs, ribosomal RNAs (rRNAs), transfer RNAs, and small nuclear RNAs, and are primarily required for maturation of rRNAs. Two main classes, box C/D snoRNAs and box H/ACA snoRNAs, have been identified. The box C/D snoRNAs and H/ACA snoRNAs guide by base pairing 2′-O-ribose methylation and pseudouridylation of specific rRNAs, respectively (reviewed in [86]). In addition to these functions, snoRNAs have been reported to have non-canonical functions such as splicing and editing (reviewed in [87]). Dysregulation of snoRNA expression has been demonstrated in various diseases including cancer (reviewed in [88]). LncRNAs have also been found to encode snoRNAs (Table 7).

**Table 7:** LncRNA encoding snoRNA and miRNA

LncRNA	snoRNA and miRNA	Human disease, species etc.	References
SNHG1	SNORD22, SNORD25, SNORD26, SNORD27, SNORD28, SNORD29, SNORD30, and SNORD31		
GAS5 (SNHG2)	SNORD44, SNORD47, SNORD74, SNORD75, SNORD76 SNORD77, SNORD78, SNORD79, SNORD80, SNORD81, and SNORA103		
SNHG3	SNORA73A and SNORA73B		
SNHG4	snoRNA U19 (SNORA74A) and SNORA74	Human	[89]
SNHG5	SNORD50A and SNORD50B	Human cancer	[90]
SNHG6	SNORD87		
SNHG7	SNORA17, SNORA17A, SNORA17B and SNORA43		
SNHG8	SNORA24		
SNHG9	SNORA78		
SNHG10	SCARNA13		
SNHG11	SNORA60 and SNORA71E		
SNHG12	SNORA16A, SNORA44, SNORA61, and SNORD99		
DANCR	SNORA26		
SNHG14	SNORD115 cluster, SNORD116 cluster, SNORD109A, and SNORD109B		
SNHG15	SNORA9		
SNHG16	SNORD1A, SNORD1B, and SNORD1C		
SNHG17	SNORA71, SNORA71A, SNORA71B, SNORA71C, and SNORA71D		
SNHG18	SNORD123		
SNHG19	snoR1 and SNORD60		
SNHG20	SCARNA16 and miR-6516		
SNHG21	SCARNA15		
SNHG22	SCARNA17 and SCARNA18		
SNHG23	SNORD113 cluster and SNORD114 cluster		
SNHG24	SNORD114 cluster		
SNHG25	SNORD104, SNORA50, SNORA50C, and SNORA76C		
MEG8	SNORD112, SNORD113-1, SNORD113-2, SNORD113-3, and miR-370		
ZFAS1	SNORD12, SNORD12B, and SNORD12C	Breast cancer	[91]

Based on University of California, Santa Cruz (UCSC) Genome Browser

LncRNA small nucleolar RNA host genes (SNHGs) encode snoRNAs, as indicated in Table 7. SNHG4 hosts small nucleolar RNA (snoRNA) U19 (SNORA74A) in intron 3 [89]. SNHG5 hosts snoRNAs, SNORD50A, and SNORD50B which were found to be deleted in various cancers, also bind KRAS protein, and inhibit RAS-ERK1/ERK2 signaling (Fig. 1C) [90]. In this study, the hosting and function of SNHG5 was not studied; however, significant tumor suppressing effects of SNORD50A and SNORD50B were demonstrated [90]. ZFAS1 which encodes SNORD12, SNORD12B, and SNORD12C functions as a tumor suppressor and is potentially a marker for breast cancer [91]. The expression level between ZFAS1 and the encoded snoRNAs was found to be not correlated [91]. There has been no report regarding functional interaction between lncRNA and its encoding snoRNA as far as we know. However, long ncRNAs with snoRNA ends (sno-lncRNAs) were discovered [92]. Sno-lncRNAs are intron-derived and have snoRNAs on both ends. The genomic region encoding one abundant class of sno-lncRNAs is specifically deleted in Prader–Willi Syndrome (PWS). SnoRNAs are known to localize to Cajal bodies or nucleoli; however, sno-lncRNAs are localized in subnuclear sites near their sites of synthesis in the PWS region. Thus, these sno-lncRNAs interact with Fox2 splicing factor and modify splicing [92]. These results indicate that cooperation of snoRNA and lncRNA enables this function.

### SnoRNA encoding miRNA

Deep sequencing has identified short RNAs derived from snoRNAs. H/ACA box snoRNAs generate RNAs of 17–19 nt in length and C/D box snoRNAs generate RNAs longer than 27 nt. SnoRNAs have been found to encode miRNAs and their expression is likely to be dependent on the host snoRNAs. A combination of deep sequencing and bioinformatics has discovered snoRNA-generated miRNAs (sno-miRNAs) (Table 8).

**Table 8:** SnoRNA encoding miRNA and piRNA

snoRNA	miRNA or piRNA	Human disease, species, etc.	References
HBII-239 (SNORD71)	miR-27b, miR-16-1, miR-28, miR-31 and let-7g	HeLa cells	[94]
ACA45	ACA45 sRNA	HEK293 cells	[93]
ACA36B (SNORA36B)	miR-664	Human	[95, 167]
ACA34	miR-1291	Human	[95, 167]
HBI-61 (SNORA81)	miR-1248	Human	[95, 167]
SNORD63	piR30840	Human CD4 primary T lymphocytes	[96]
SNORD44	pi-sno44	Breast cancer	[97]
SNORD74	pi-sno74	Breast cancer	[97]
SNORD75	pi-sno75	Breast cancer	[97]
SNORD78	pi-sno78	Breast cancer	[97]
SNORD81	pi-sno81	Breast cancer	[97]

Generation of miRNA AKA45S from the H/ACA box snoRNA ACA45 was found to be DICER-dependent and AKA45S potentially targets the mRNA of Cyclin-Dependent Kinase 19 (CDC2L6) in HEK293 cells [93]. This suggests that AKA45S functions as an miRNA [93]. Computational analyses also identified 84 miRNAs that box C/D snoRNAs or their precursors encode, and have similarity to box C/D snoRNAs. Of these miRNAs, C/D box snoRNA HBII-239 (SNORD71)-derived miRNA precursors, miR-27b, miR-16-1, mir-28, miR-31, and let-7g were found to bind to fibrillarin protein, a component of a nucleolar small nuclear ribonucleoprotein (snRNP), suggesting that these miRNAs evolved from the snoRNA (Fig. 1D) [94]. Deep sequencing revealed that C/D box snoRNAs generated sno-miRNAs [95]. Experimental analysis shows that C/D snoRNAs, ACA36b, HBI-61, and ACA34, encode sno-miRNAs, miR-664, miR-1248, and miR-1291, respectively, which function in gene silencing [95]. These studies indicate that miRNAs may have evolved from snoRNAs and gained their functions.

### SnoRNA encoding Piwi-interacting RNA

Piwi-interacting RNAs (piRNAs), 24–31 nucleotides in length, are the largest class of small non-coding RNA molecules expressed in animal cells. PiRNAs form silencing complexes by interacting with piwi proteins, a subfamily of the Argonaute proteins, and the complexes repress transposons and other genetic elements in germ line cells, especially genetic elements in spermatogenesis through transcriptional or post-transcriptional mechanisms. A combination of deep sequencing and bioinformatics also has discovered snoRNA-generated piRNAs (sno-piRNAs) (Table 8).

Two studies have notably demonstrated that snoRNA-derived piRNAs are functional in human cells. Deep sequencing has identified 20 C/D snoRNAs-derived piRNAs (sno-piRNAs) in human CD4 primary T lymphocytes [96]. One sno-piRNA, piR30840, downregulates the expression of IL-4 by binding to the pre-mRNA intron of IL4 in a sequence-specific manner [96]. This downregulation was found to be associated with Piwil4 and Ago4, which further interacts with Trf4–Air2–Mtr4 Polyadenylation (TRAMP) complex [96]. Another study has shown that lncRNA GAS5 hosts several snoRNAs and some of them generate sno-piRNAs. One of these, pi-sno75 upregulates the transcription of tumor necrosis factor (TNF)-related apoptosis inducing ligand (TRAIL), a proapoptotic protein, by binding to its promoter. Pi-sno75 interacts with the PIWIL1*/*4 proteins and recruits the MLL3/hCOMPASS complex to the promoter region of TRAIL which leads to H3K4 methylation and H3K27 demethylation and induces TRAIL mRNA (Fig. 1E) [97]. Interestingly, pi-sno75 upregulates TRAIL by binding to its promoter; however, it may also have suppression effects on other genes.

The other study has shown that small RNA sequencing of breast cancer and normal breast tissues revealed snoRNAs which were potential biomarkers for overall survival and/or recurrence-free survival. Six snoRNAs were found to host miRNAs and 48 snoRNAs were found to host piRNAs that potentially target oncogenes in breast cancer [98]. These studies have indicated that piRNAs are generated from snoRNA and these snoRNA-derived piRNAs are functional. Study in sno-piRNA is in an early stage and further investigations are awaited.

## Conclusion and perspectives

RNA sequencing of whole genomes has revealed that ncRNAs constitute 98% of the human genome. Originally, the majority of DNA and ncRNAs were thought to be non-functional. The discovery of functional roles for these ncRNAs has been remarkable. Networking between ncRNAs has opened a complex regulatory world, adding to their significance in the regulation of diverse biological functions. Integration of networks of protein, non-coding, and coding RNA is of great importance requiring further investigation. Here, we described some of the interactions and cross-talk between ncRNAs.

MiRNA–lncRNA interaction including ceRNA studies has been done using overexpression or knockdown of lncRNA or miRNA. These methods disrupt physiological conditions in cells. Any overexpressed lncRNA may trigger ceRNA regulation or serve as a target of miRNAs because of high copy numbers. In siRNA knockdown, the high concentration of siRNA could bind to off-targets and also perturb the RISC complex. The CRISPR/Cas9-gRNA complex system, a recently developed genome editing system, could disrupt miRNA-binding sites and be used for miRNA–lncRNA interaction studies with a precise targeting, although no experimental method is without its drawbacks.

It has been reported that changes in ceRNAs are too small to influence miRNA-mediated repression, since the number of MREs is too high for the number of targeting miRNAs using miR-122 targets in hepatocytes and livers as a model [99, 100]. It is questionable that a single miR-122 study may be used to generalize to ceRNA regulation. Another study using Ago2 individual-nucleotide resolution UV crosslinking and immunoprecipitation (iCLIP) has demonstrated that highly expressed miRNA families such as miR-294 and let-7 are likely not function in ceRNA mechanisms because of the high miRNA and target concentrations [101]. In contrasted to miR-294 and let-7, high-affinity lncRNA targets of miRNAs of low abundance such as miR-92/25 may function as ceRNAs physiologically [101]. This study is more likely applicable to ceRNA regulation. The ratio between the number of miRNA-binding sites and miRNA molecules is essential for ceRNA regulation and changing the ratio could trigger ceRNA regulation. Since the expression of ncRNAs is specifically regulated with regard to time and location, the changes in cellular status such as disease incidence, development, or stimulation from extracellular sources cause the changes in ncRNA expression and trigger ceRNA regulation.

A number of reports using clinical samples demonstrate that inverse expression levels between lncRNAs and miRNAs which may interact with each other, which suggest that these interactions and targeting take place at physiological levels, although no direct evidence has been presented. Various studies with clinical samples also indicate that ceRNA regulation may occur physiologically, which also has not been directly proven. As discussed in “Overlapping of ceRNA regulatory mechanisms and downregulation of lncRNA by direct miRNA–lncRNA interactions”, the interactions between lncRNAs and miRNAs which mediate ceRNA mechanisms may partially overlap with those of miRNAs and lncRNAs triggering lncRNA decay of or inhibiting lncRNA function. This possible overlap may prevent precise explanation of these experimental data.

MiRNAs are generated in the cytoplasm, bind to the 3′UTR of mRNAs in a sequence-dependent manner, and trigger decay of the mRNAs. In addition to this cytoplasmic activity, miRNAs have been shown to interact with lncRNAs in both the cytoplasm and nucleus. The interaction between miRNA and lncRNA may depend on the concentration of these non-coding RNAs in specific subcellular compartments. These interactions need to be investigated in mouse models for resolving these unsolved issues at physiological levels. Identification of miRNA–target interactions in specific cellular compartments using techniques such as HITS-CLIP and PAR-CLIP and integration of these data with computational analysis would lead to elucidation of ceRNA function and how the intertwined network is regulated.

There have been studies for other ncRNA types. Interestingly, oncogenic lncRNA MALAT1 yields a small tRNA-like cytoplasmic RNA by 3′ end processing [103]. tRNA-derived small RNAs have been reported to significantly silence RNAs by associating with Argonautes 3 and 4 [104]. These studies indicate that tRNA may have gene suppression effects in cancer cells. SnoRNA HBII-52 (SNORD115) has been shown to regulate splicing by binding to the serotonin receptor 2C mRNA [105]. Ultraconserved regions (T-UCRs) are consistently and significantly altered at the genomic level in leukemias and carcinomas, and miRNAs may interact with T-UCRs with significant antisense complementarity [106]. Other small RNAs such as Y RNA, spliced leader RNA (SL RNA), etc. may target other mRNAs and ncRNAs, regulate their expression, and form regulatory networks. Further investigation in the expanding field of ncRNA interaction will reveal its biological function and regulation, and may lead to therapeutic strategies for cancers and other diseases.
